# A randomized, open-label study to investigate the effect of belimumab on pneumococcal vaccination in patients with active, autoantibody-positive systemic lupus erythematosus

**DOI:** 10.1177/0961203317703495

**Published:** 2017-05-03

**Authors:** W Chatham, A Chadha, J Fettiplace, C Kleoudis, D Bass, D Roth, D Gordon

**Affiliations:** 1 University of Alabama at Birmingham, Birmingham, USA; 2 Austin Regional Clinic, Austin, USA; 3 GSK, Uxbridge, Middlesex, UK; 4 Parexel, Raleigh-Durham, USA; 5 GSK, Collegeville, USA

**Keywords:** Pneumococcal, vaccination, belimumab, systemic lupus erythematosus

## Abstract

**Objective:**

Intravenous belimumab 10 mg/kg is approved as an add-on therapy in patients with active, autoantibody-positive systemic lupus erythematosus. This study aimed to assess the impact of belimumab on immune response to pneumococcal vaccination in patients with systemic lupus erythematosus.

**Methods:**

This was a Phase 4, open-label study (GSK BEL115470; NCT01597492) conducted in the United States. Patients were randomized (7:9) to receive a 23-valent pneumococcal vaccination four weeks prior to (pre-belimumab cohort) or 24 weeks after (belimumab-concurrent cohort) commencing four-weekly belimumab 10 mg/kg intravenous treatment plus standard systemic lupus erythematosus therapy. Analyses of vaccine titers were performed on the as-treated population (received ≥1 dose of belimumab). The primary endpoint was the proportion of patients with positive antibody responses (≥2-fold increase from pre-vaccination levels, or post-vaccination level ≥ 0.6 µg/mL if pre-vaccination levels were unquantifiable) to ≥1 of 23 pneumococcal vaccine serotypes, four weeks post vaccination. Other endpoints included the proportion of patients with positive antibody responses to ≥2 to ≥10, and ≥11–23 (post hoc analysis) of serotypes. Safety was assessed by monitoring adverse events.

**Results:**

Seventy-nine patients received pneumococcal vaccination (pre-belimumab cohort, *n* = 34; belimumab-concurrent cohort, *n* = 45). The majority (87.3% [69/79]) completed the study; 10 (12.7%) withdrew (patient request, *n* = 3; adverse event, *n* = 3; lost to follow-up, *n* = 2; other, *n* = 2). At Week 4 post-vaccination, 97.0% (32/33) and 97.6% (40/41) of patients (pre-belimumab and concurrent belimumab cohorts, respectively) had a positive response to ≥1 of 23 pneumococcal serotypes. Over 85% of patients in both cohorts responded to ≥10 of serotypes, approximately 80% responded to ≥12 serotypes, and approximately two-thirds responded to ≥16 serotypes. Little difference was observed between cohorts across a broad response, up to 23 serotypes. Eight (23.5%) patients experienced an adverse event considered by the investigator to be treatment-related in the pre-belimumab cohort and four (8.9%) in the belimumab-concurrent cohort; seven patients experienced non-fatal serious adverse events (pre-belimumab cohort, 11.8% [*n* = 4]; concurrent-belimumab cohort, 6.7% [*n* = 3]), and no deaths were reported.

**Conclusion:**

The proportion of patients generating a response to ≥1 pneumococcal serotype did not differ between the pre-belimumab and belimumab-concurrent cohorts; the proportions were also comparable across a broader response (from ≥2 serotypes to 23 serotypes).

## Introduction

Patients with systemic lupus erythematosus (SLE) are more susceptible to infections, which are a major cause of morbidity and mortality in this population.^1–3^ Although patients with SLE can have impaired responses to immunization, vaccines still play an important role in protection against numerous infections, including influenza and pneumonia.^4,5^ Impaired responses to immunization in patients with SLE could be further hampered by concomitant immunosuppressive medications.^6^ A previous study suggested that patients with SLE who were treated with immunosuppressives had diminished responses to influenza vaccination, but the use of concomitant antimalarials appeared to mitigate the diminution.^6^ In accordance with European League Against Rheumatism recommendations, adult patients with autoimmune inflammatory rheumatic diseases should be strongly considered for a 23-valent polysaccharide pneumococcal vaccination and a tetanus toxoid vaccination should be administered in accordance with the recommendation for the general population.^7^

Belimumab is a recombinant, human, immunoglobulin (IgG1λ) monoclonal antibody, licensed for the treatment of adult patients with active, autoantibody-positive SLE who are receiving standard SLE therapy (SoC).^8^ Belimumab binds to and inhibits the biological activity of soluble B-lymphocyte stimulator (BLyS) protein, which is critical in the survival and differentiation of B cells.^9^ Belimumab has been shown to significantly reduce circulating CD19+, CD20+, naïve, activated B cells, and plasma cells, while causing a transient increase in memory cells that returns to baseline levels by Week 52.^10^ Theoretically, by neutralizing the activity of BLyS, belimumab inhibits the maturation and survival of B cells and thus may affect responses to vaccines.^11^ In a vaccine sub-study of BLISS-76 (NCT00410384), a large, randomized Phase 3 study of belimumab in patients with SLE,^12^ belimumab treatment did not affect pre-existing pneumococcal antibody responses in patients with SLE, suggesting that belimumab does not affect long-lived plasma and memory B cells.^13^

Data regarding the effect of belimumab on immune responses to vaccination are limited. However, one previous study suggested that belimumab use in SLE in combination with traditional disease-modifying antirheumatic drugs or prednisolone did not impair antibody response after vaccination with a 13-valent polysaccharide pneumococcal vaccination.^14^ The original objective of the current study was to assess the impact of belimumab on immune response to pneumococcal and tetanus vaccination in patients with SLE.

## **Methods**

### Study design

This Phase 4, randomized, open-label study (GSK study BEL115470; NCT01597492) was conducted at 13 centers in the United States (US) between May 2012 and November 2015. The study was approved in accordance with the International Conference on Harmonisation of Technical Requirements for Registration of Pharmaceuticals for Human Use Good Clinical Practice^15^ and was conducted in accordance with the Declaration of Helsinki 2008^16^ and applicable country-specific requirements.

Patients were vaccinated with a 23-valent pneumococcal vaccine (Pneumovax®23, Sanofi Pasteur), either prior to commencing treatment with belimumab (pre-belimumab cohort) or at Week 24 of belimumab treatment (belimumab-concurrent cohort). To account for the expected difference in dropout rates at Week 4 and Week 28 (four weeks post vaccination for each group), patients were unequally randomized in a 7:9 ratio. Patients received open-label belimumab 10 mg/kg intravenously (IV) at Weeks 4, 6, and 8 and then every four weeks to Week 32 (nine doses, pre-belimumab cohort) or at baseline and Weeks 2 and 4, and then every four weeks to Week 28 (nine doses, belimumab-concurrent cohort). The baseline visit was defined as the latest pneumococcal titer collected prior to vaccination (Week 0 for both cohorts; Week 24 for the belimumab-concurrent cohort following a protocol amendment). Post-vaccination pneumococcal titers were assessed at Week 4, prior to the first dose of belimumab, in the pre-belimumab cohort and at Week 28, prior to the last dose of belimumab, in the belimumab-concurrent cohort. Exit visits occurred on Week 32 and Week 28 for the pre-belimumab and belimumab-concurrent cohorts, respectively.

After commencement of the study, the study protocol was amended to remove the administration and assessment of tetanus vaccine response, and the study therefore focused on pneumococcal vaccination alone. In addition, due to difficulties in recruiting patients without pre-existing pneumococcal titers, a protocol amendment increased the number of permitted pre-existing pneumococcal antibody titers (>1.0 µg/mL) at baseline from ≤4 of 23 to ≤7 and then ≤9 of 23, to facilitate recruitment.

### Study population

All patients provided written informed consent. Patients included were ≥18 years of age, with a clinical diagnosis of SLE according to the American College of Rheumatology criteria,^17,18^ and active autoantibody-positive SLE, defined as the presence of anti-nuclear antibody (ANA; positive titer ≥1:80) and/or anti-double-stranded DNA (dsDNA) antibodies (≥30 IU/mL). Patients were receiving SoC at screening and had pre-existing pneumococcal antibody titers (>1.0 µg/mL) to ≤4, ≤7 or ≤9 of 23 vaccine serotypes, depending on the point at which patients were enrolled in the study, due to protocol amendments, as detailed above. Patients were excluded from enrollment if they had previously received any prior treatment with belimumab, or treatment with any biologic within 364 days of Day 0, except for the following, which were excluded if received within 90 days of Day 0: anti-tumor necrosis factor therapy, interleukin-1 receptor antagonist, plasmapheresis, corticosteroids at doses ≥100 mg/day prednisone (or equivalent), or IV cyclophosphamide. Patients in receipt of a non-biologic investigational agent within 60 days of Day 0, corticosteroids at doses ≥40 mg/day prednisone (or equivalent), live vaccine within 30 days of Day 0, pneumococcal vaccination within five years of screening, or who had ever had a hypersensitivity reaction to an immunization were also excluded.

### Primary endpoint

The primary endpoint of the study was the proportion of patients with positive antibody responses to ≥1 of the 23 pneumococcal vaccine serotypes measured four weeks post vaccination. A positive antibody response was defined as a two-fold increase from pre-vaccination levels against ≥1 of the 23 pneumococcal serotypes measured. For patients with unquantifiable pre-vaccination levels, a positive antibody response was considered to be a post-vaccination level of ≥0.6 µg/mL (≥2-fold increase above the lower limit of quantification [≥0.3 µg/mL] for this assay).

### Other endpoints

The proportion of patients with positive antibody responses to ≥11 to 23 of the serotypes was analyzed post hoc, as a response to ≥16 (70%) of serotypes is thought to be clinically relevant.^19^ The proportion of patients with positive antibody responses to ≥2, ≥3, ≥4, ≥5, ≥6, ≥7, ≥8, ≥9, and ≥10 of the 23 pneumococcal serotypes four weeks post vaccination was a pre-specified vaccine response endpoint.

A subset analysis of the primary endpoint was performed for exploratory purposes, to examine the effects of concomitant medications on vaccine response, for the following subgroups: baseline antimalarial use (yes, no); baseline corticosteroid dose (>7.5 mg/day vs ≤7.5 mg/day), and baseline immunosuppressive use (any vs none). This subset analysis was also performed post hoc for patients with a response to ≥12 of the serotypes.

Safety was evaluated by monitoring of adverse events (AEs), clinical laboratory tests, immunogenicity, physical examinations, and vital signs (temperature, sitting systolic and diastolic blood pressure, and heart rate) through to ≥8 weeks post treatment. Pre-treatment AEs were defined as AEs that occurred prior to the first dose of belimumab and, in the pre-belimumab cohort, AEs that occurred after vaccination but before the first belimumab infusion. Treatment-emergent AEs were defined as an AE that emerged on or after the first dose of belimumab, having been absent pre-treatment, or that worsened relative to the pre-treatment state. Any AEs with partial or missing start and/or stop dates were assumed to be treatment-emergent unless comparison of partial dates suggested otherwise.

### Analyses and analysis populations

As this was an open-label study, statistical analyses of the endpoints were exploratory in nature.

Statistical outputs were produced using the intent-to-treat (ITT) population, defined as all patients who were randomized and received ≥1 dose of vaccine and/or belimumab infusion. All summaries using the ITT population grouped patients according to the randomized treatment, regardless of the treatment received. All analyses of safety data were performed on the ITT population. The as-treated population was defined as all patients who received ≥1 dose of belimumab. All analyses of vaccine titer were performed on this population, and grouped patients according to the treatment administered. As all patients received ≥1 dose of belimumab, the ITT and as-treated populations were identical in this study. Immunogenicity assessments were performed using a binding assay and neutralizing assay. The aim was to achieve ≥30 evaluable patients (four weeks post vaccination) in each cohort in a 1:1 ratio. A sample size of 30 patients allowed for the rate of response to ≥1 pneumococcal serotype to be estimated with an exact 95% CI of 37.7–74.8%, assuming a response rate of 57%.^20^

## Results

### Study population

**Figure 1:**
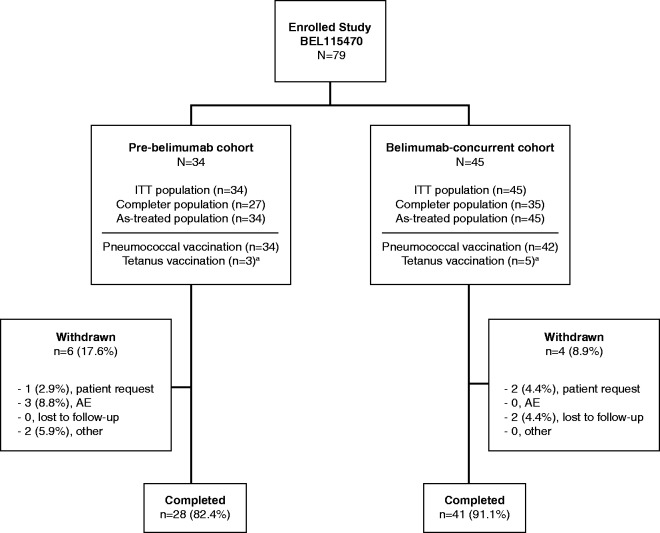
Patient disposition and enrollment. ^a^ Patients recruited prior to protocol change removing the administration and analysis of tetanus vaccination. AE: adverse event; ITT: intent-to-treat.

A total of 79 patients were enrolled and randomized to receive pneumococcal vaccination in the pre-belimumab (*N* = 34) and belimumab-concurrent (*N* = 45) cohorts (ITT population; Figure 1). All patients received ≥1 dose of belimumab (as-treated population). The majority of patients (87.3% [69/79]) completed the study. Ten patients withdrew; three at the patient’s request, three due to AEs, two were lost to follow-up, and a further two for other reasons. There were 27 patients in the pre-belimumab cohort and 35 patients in the belimumab-concurrent cohort who completed all nine doses of the planned treatment period. The majority of patients were female (91.1% [72/79]) and the mean age was 39.6 years. Patients were predominantly White (65.8% [52/79), while Black or African American patients comprised 25.3% (20/79) of the population (Table 1). The majority of patients received a combination of SLE therapies; 45.6% (36/79) were on immunosuppressants at baseline, either alone or in combination.

**Table 1:** Baseline patient characteristics (ITT population)

Characteristic	Pre-belimumab cohort *N* = 34	Belimumab-concurrent cohort *N* = 45	Total *N* = 79
Female, *n* (%)	29 (85.3)	43 (95.6)	72 (91.1)
Age (years), mean (SD)	41.0 (12.57)	38.6 (12.31)	39.6 (12.40)
BMI (kg/m^2^), mean (SD)	29.4 (7.40)	30.0 (8.87)	29.7 (8.23)
Race, *n* (%)			
White	25 (73.5)	27 (60.0)	52 (65.8)
Black or African American/African heritage	8 (23.5)	12 (26.7)	20 (25.3)
Other	1 (2.9)	6 (13.3)	7 (8.9)
SLE disease duration (years), mean (SD)^a^	7.9 (8.71)	7.6 (7.36)	7.7 (7.92)
Duration of exposure (days), mean (SD)^b^	205.9 (53.47)	219.8 (23.26)	213.8 (39.54)
Total number of infusions per patient, mean (SD)	8.1 (2.06)	8.6 (0.83)	8.4 (1.50)
SLE medication usage, *n* (%)			
Steroid and immunosuppressant and antimalarial only	12 (35.3)	14 (31.1)	26 (32.9)
Antimalarial only	5 (14.7)	14 (31.1)	19 (24.1)
Steroid and antimalarial only	7 (20.6)	10 (22.2)	17 (21.5)
Immunosuppressant and antimalarial only	4 (11.8)	2 (4.4)	6 (7.6)
Steroid and immunosuppressant only	0	3 (6.7)	3 (3.8)
Steroid only	2 (5.9)	0	2 (2.5)
Immunosuppressant only	1 (2.9)	0	1 (1.3)
Average daily corticosteroid dose, mean (SD)	6.5 (8.73)	7.1 (7.95)	6.9 (8.25)
0 mg/day, *n* (%)	13 (38.2)	18 (40.0)	31 (39.2)
>0 to ≤7.5 mg/day, *n* (%)	9 (26.5)	6 (13.3)	15 (19.0)
>7.5 mg/day, *n* (%)	12 (35.3)	21 (46.7)	33 (41.8)

BMI: body mass index; ITT: intent-to-treat; SD: standard deviation; SLE: systemic lupus erythematosus.

a Duration defined as screening date to SLE diagnosis date plus one day.

b Duration of exposure defined as the last infusion date to the first infusion date plus 28 days. Only complete dates were used; first and last infusion dates were used, regardless of any missed doses.

### Vaccine response

**Figure 2:**
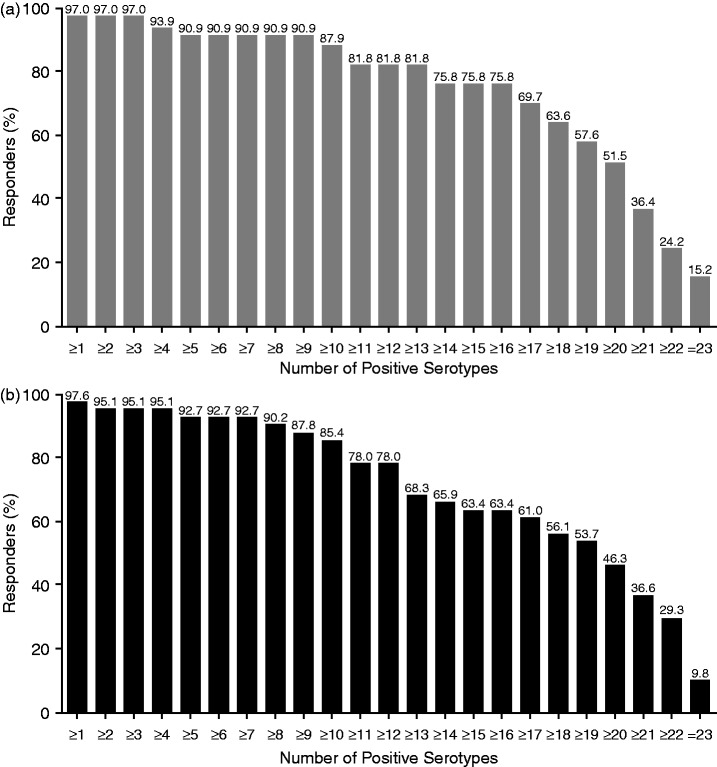
Pneumococcal positive vaccine response to number of serotypes (observed; as-treated population). (a) Pre-belimumab cohort and (b) Belimumab-concurrent cohort.

At four weeks post vaccination, 97.0% (32/33) of patients in the pre-belimumab and 97.6% (40/41) in the belimumab-concurrent cohorts demonstrated a positive response to ≥1 of the 23 pneumococcal serotypes measured (as-treated population; Figure 2). Among the two patients who did not respond to the pneumococcal vaccine, one patient (pre-belimumab cohort) had low baseline IgG levels (normal 6.94–16.18 g/L) that did not improve during the study (baseline, 5.39 g/L; Week 24, 5.16 g/L) and one patient (belimumab-concurrent cohort) had normal IgG levels throughout the study (baseline, 8.13 g/L; Week 24, 7.89 g/L). Over 85% of patients in both cohorts achieved a positive response to ≥10 of the 23 pneumococcal serotypes. In post hoc analyses, a comparable proportion of patients in the pre-belimumab and the belimumab-concurrent cohorts attained protective immunity to 50% (≥12/23) of serotypes (81.8% [27/33] and 78.0% [32/41], respectively) and 70% (≥16/23) of serotypes (75.8% [25/33] and 63.4% [26/41], respectively).

One patient in each cohort did not achieve a positive response to ≥1 of the 23 pneumococcal serotypes; these patients were both taking a concomitant antimalarial and immunosuppressant at baseline, and their baseline corticosteroid dose was ≤7.5 mg (Table 2). In post hoc analyses, in the pre-belimumab cohort the proportion of patients who had a response to ≥12 serotypes appeared higher among patients receiving a corticosteroid dose of >7.5 mg/day at baseline versus those receiving >0 to ≤7.5 mg/day (90.9% [10/11] vs 77.3% [17/22]); in the concurrent belimumab cohort the proportions were comparable. In both cohorts, more patients who did not receive immunosuppressives at baseline appeared to have a response to ≥12 serotypes, compared with those who did receive immunosuppressives (Table 2).

**Table 2:** Positive response to ≥1 and ≥12 pneumococcal vaccine serotype by baseline medication use (as-treated population)

Baseline medication subgroup	Pre-belimumab cohort *N* = 34	Belimumab-concurrent cohort *N* = 45
Number of patients responding to ≥1 pneumococcal vaccine serotype, *n*/*N* (%)		
Antimalarial use		
Yes	26/27 (96.3)	36/37 (97.3)
No	6/6 (100.0)	4/4 (100.0)
Corticosteroid dose		
>0 to ≤7.5 mg/day	21/22 (95.5)	21/22 (95.5)
>7.5 mg/day	11/11 (100.0)	19/19 (100.0)
Immunosuppressive use		
Any	16/17 (94.1)	17/18 (94.4)
None	16/16 (100.0)	23/23 (100.0)
Number of patients responding to ≥12 pneumococcal vaccine serotype, *n*/*N* (%)		
Antimalarial use		
Yes	23/27 (85.2)	28/37 (75.7)
No	4/6 (66.7)	4/4 (100.0)
Corticosteroid dose		
>0 to ≤7.5 mg/day	17/22 (77.3)	17/22 (77.3)
>7.5 mg/day	10/11 (90.9)	15/19 (78.9)
Immunosuppressive use		
Any	12/17 (70.6)	13/18 (72.2)
None	15/16 (93.8)	19/23 (82.6)

### Safety

The proportion of patients experiencing ≥1 AE was 91.2% (31/34) in the pre-belimumab cohort and 86.7% (39/45) in the belimumab-concurrent cohort, and the most common AEs were arthralgia and nausea (Supplementary table). The proportion of patients with an AE considered by the investigator to be treatment-related was 23.5% (8/34) for the pre-belimumab cohort and 8.9% (4/45) for the belimumab-concurrent cohort. Two patients (4.4%) in the belimumab-concurrent cohort experienced a total of four AEs that were judged by the investigator to be at least possibly vaccine-related (one [2.2%] each of arthralgia, myalgia, nausea, and pyrexia). Four (11.8%) patients in the pre-belimumab cohort and three (6.7%) patients in the belimumab-concurrent cohort experienced non-fatal serious AEs. Three patients in the belimumab-concurrent cohort had a suicide-related AE, two of which were serious (one suicide attempt and a self-hospitalization to a psychiatric facility). There were no suicide-related cases in the pre-belimumab cohort. No malignant neoplasms or deaths were reported, and no AE was recorded more than once for each patient. There were no safety concerns based on the results of laboratory tests, immunogenicity assessments, physical examinations, and vital signs.

### Biomarkers

In post hoc analyses, baseline IgA, IgG, and IgM levels were analyzed in patients responding to more than (responders, *n* = 59) or less than (non-responders, *n* = 15) 50% of serotypes. Mean (standard deviation) Ig levels appeared to be slightly lower in the non-responders (IgA 2.60 [1.630] g/L; IgG 11.63 [4.899] g/L; IgM 0.94 [1.165] g/L) compared with the responders (IgA 2.85 [1.177] g/L; IgG 14.56 [5.731] g/L; IgM 1.16 [0.885] g/L), though levels were not deemed to be low in either group. Among non-responders, 3/15 (20.0%) patients had baseline IgG levels < 7 g/L compared with 2/59 (3.4%) responders.

## Discussion

This randomized, open-label study investigated pneumococcal vaccine response in patients before and after commencing belimumab treatment. It was demonstrated that the proportion of patients generating a response to ≥1 serotype was very high, and did not differ between those who were vaccinated prior to commencing belimumab and those who were vaccinated after receiving treatment with belimumab for 24 weeks. Similarly, little difference was observed across a broader response (from ≥2 serotypes up to 23 serotypes).

The primary endpoint of this study, which was created in collaboration with regulatory agencies, was the proportion of patients with a response to ≥1 serotype, and other endpoints included the proportion of patients responding to a broader range of up to ≥10 serotypes. Although not clinically validated, expert panel recommendation is that an adequate response to 70% and 50% of serotypes tested (equivalent to ≥16 and ≥12 serotypes in this study) is a normal response for those over five years of age, and children aged two to five years, respectively.^19,21,22^ Thus, post hoc analyses were carried out in this study to investigate additional cut-off levels. The majority of patients responded to 50% and 70% of serotypes, and results appeared to be comparable between the pre-belimumab and concurrent-belimumab cohorts.

In further post hoc analyses, mean baseline Ig levels (IgA, IgG and IgM) in the 15 patients who did not respond to 50% of serotypes were not deemed as ‘low,’ suggesting that these patients were not immunodeficient. However, a higher percentage (20%) of non-responders had low IgG (<7 g/L) compared with responders (3.4%), and mean Ig levels appeared to be slightly lower in the non-responders compared with responders. Low or low-normal range Ig levels in patients with SLE may be due to SLE developing in the context of underlying humoral immune deficiency.^23^ Indeed, the assessment of responses to pneumococcal vaccination is often employed to identify such patients in clinical practice. Low Ig levels in patients with SLE may also be due to intercurrent immunosuppressive therapy. Studies investigating immunological therapies have previously demonstrated the potential for attenuation of patient vaccination responsiveness, and there is evidence that concomitant medications may affect the outcome. One study investigating the effects of rituximab and concomitant methotrexate on vaccination responses in patients with rheumatoid arthritis showed a decreased response to pneumococcal polysaccharide vaccine in patients receiving rituximab compared with patients treated with methotrexate alone.^20^ In a study of patients with SLE, the response to influenza vaccine was reduced by immunosuppressive therapies, but appeared to be restored by antimalarial treatment.^6^ In the present study, no trend was identified in terms of baseline medication use and a response to ≥1 serotype. The proportion of patients with a response to ≥12 serotypes appeared to be higher in both cohorts among patients who did not receive immunosuppressives at baseline compared with those who did. However, patient numbers were small in these analyses.

The results of this study are consistent with a small sub-study of the Phase 3 BLISS-76 trial, which evaluated the effects of belimumab on pre-existing antibody levels against pneumococcal, tetanus, and influenza antigens in patients with SLE.^13^ In this sub-study, a sub-population of patients received pneumococcal (*n* = 7) vaccination. All patients treated with belimumab or placebo who received pneumococcal vaccination (*n* = 7, five of whom received belimumab) were able to mount a protective response and responses did not differ between treatment groups. However, patient numbers in the BLISS 76 sub-study were too small to draw conclusions.^13^

The safety profile observed in this study was acceptable and generally reflected the established safety profile of belimumab;^24^ however, in this study there is no placebo group for comparison. Two serious suicide/self-injury events (one suicide attempt and a self-hospitalization to a psychiatric facility) were reported in the belimumab-concurrent cohort (none were reported in the pre-belimumab cohort), but there were no completed suicides. The overall incidence of these events was 2.5%, which is higher than observed in previous, larger studies, but may be a chance observation given the size of the study. The potential for a relationship between suicide-related events and belimumab is being formally addressed by two large ongoing prospective studies (BEL116543 [NCT01729455] and BEL115467 [NCT01705977]), designed to capture more detailed information about psychiatric events and other AEs of special interest.

The limitations of this study include the lack of a placebo arm and the small sample size. Also, less than 50% of patients who were enrolled were taking concurrent immunosuppressants that could potentially affect vaccine responses when belimumab is added to their treatment regimen.

Infections are a major cause of mortality in patients with SLE.^2,3,5,6^ This study provides additional data supporting the notion that treatment with belimumab does not appear to affect responses to pneumococcal vaccination in patients with SLE. Furthermore, the results of this study theoretically indicate that, in general, vaccination responses in patients with SLE may not be negatively affected by belimumab.

## Supplementary Material

Supplementary material
