# Supplementary material for: A randomized, open-label study to investigate the effect of belimumab on pneumococcal vaccination in patients with active, autoantibody-positive systemic lupus erythematosus
Source: Lupus. 2017 May 3;26(14):1483–90. doi: 10.1177/0961203317703495 (PMC5673008; doi:10.1177/0961203317703495)
Supplement: Supplementary material [file LUP703495_supplementary_material.pdf]

Supplementary table: Summary of AEs (ITT population)

AEs, n (%)	Pre-belimumab cohort N=34	Belimumab-concurrent cohort N=45
**Any AE[a]**	31 (91.2)	39 (86.7)
Arthralgia	6 (17.6)	10 (22.2)
Nausea	6 (17.6)	9 (20.0)
Viral gastroenteritis	1 (2.9)	6 (13.3)
Cough	4 (11.8)	3 (6.7)
Dizziness	4 (11.8)	2 (4.4)
Diarrhea	3 (8.8)	5 (11.1)
**Treatment-related AE**	8 (23.5)	4 (8.9)
**Vaccine-related AE**	0	2 (4.4)
**Any SAE**	4 (11.8)	3 (6.7)
**Severe AE**	8 (23.5)	9 (20.0)
**Serious and/or severe AE**	9 (26.5)	10 (22.2)
**AE resulting in study agent discontinuation**	3 (8.8)	0
**Death**	0	0

[a]AEs reported in ≥10% of patients in either cohort are listed.
AE, adverse event; ITT, intent-to-treat; SAE, serious adverse event.
